# “Self-care is being attentive to yourself”: using assemblages to examine discursive-material practices of self-care among Filipino university students

**DOI:** 10.1080/17482631.2023.2247619

**Published:** 2023-10-02

**Authors:** Gilana Kim T. Roxas

**Affiliations:** Department of Psychology, Ateneo de Manila University, Quezon City, Philippines

**Keywords:** Assemblage, mental health, self-care, photo elicitation, pandemic, university students, Philippines

## Abstract

**Purpose:**

Researchers have observed a rise in mental health issues among university students over the course of the pandemic, in part due to the closure of schools and public spaces for wellness. Thus, the purpose of this study is to explore how students creatively reassemble their self-care practices through different objects and spaces within their homes to care for their mental health.

**Methods:**

Photo-elicitation interviews were conducted with ten (10) female university students from the Philippines. Photographs and interview transcripts were analysed through map-making practices and an iterative process of crafting assemblages and their cofunctionings.

**Results:**

The work-home assemblage was identified as the site of self-care, delineated by boundary-making and place-making practices and the creation of time spaces. The two major cofunctionings of the work-home assemblage were (a) caring for the self as fostering different relationalities with the self, and (b) caring for the self as copresencing with human and nonhuman others.

**Conclusion:**

Findings demonstrate the importance of material and spatial relationalities in facilitating caring relational encounters with the self. Theoretical and practical implications include attuning to the existing material and spatial resources in one’s environment and integrating them into one’s practices of caring for the self.

## Introduction

The novel Coronavirus disease (COVID-19) was the largest global public health challenge of the century since its outbreak in Wuhan, China in December 2019 (World Health Organization, 2020). As a context, the pandemic has produced multiple space-time disruptions in the regular flow of activity in society, leaving those who dedicate most of their hours in workplaces or educational institutions vulnerable to the psychological impact of the pandemic (Duong et al., 2020). In response, experts have recommended self-care practices to cope with the psychological distress of stay-at-home orders (Huang, 2020). In fact, there was a marked increase in searches for self-care worldwide compared to searches for formal mental health services during the pandemic (Knipe et al., 2020).

In this study, I examine Filipino university students’ assemblages of self-care during the pandemic. Among the Filipinos surveyed in the general population, it was found that Filipino students had been particularly vulnerable to the psychological impact of the pandemic compared to Filipinos who were employed (Tee et al., 2020), likely due to the abrupt shift to online modes of learning and its accompanying ramifications (e.g., faulty internet connection, lack of social support, etc.) (Hasan & Bao, 2020). Additionally, there were prolonged negative effects on the mental health on Filipino students given that the Philippines had one of the longest lockdowns in the world (See, 2021), which made the country lag behind others in resuming face-to-face classes (UNICEF, 2021). In one survey, nearly half of young Filipino girls and young women reported being worried about their “slim chances of returning to school” (Plan International, 2020).

Given these, I situate my inquiry of self-care in the fluid coming together of assemblages—discursive-material elements and processes of caring for one’s psychosocial health. I apply assemblage thinking to examine the spatiotemporal contingent ways of self-care among Filipino university students given the disruptions produced by the COVID-19 pandemic. This study contributes to the existing materialist strand in psychological literature by empirically demonstrating how photographs and narratives may be used as multiple entryways into participants’ experiences. Such findings illuminate that self-care is a relational, embodied, and emplaced event, where participants’ connectivities with different discursive-material configurations facilitate healing encounters with themselves and enable affective experiences of health and wellbeing.

### Challenging dominant models of self-care

Despite the surge of popularity in talk on self-care in recent years, scholarly literature on self-care remains largely fragmented (Godfrey et al., 2011; Jiang et al., 2020), in part because the term self-care is shared across the disciplines of medicine, public health, and psychology. For example, in the medical and allied health disciplines, self-care is conceptualized as a set of behaviours pertaining to the maintenance, monitoring, and management of a chronically ill, disabled, or recovering body (Riegel et al., 2012). In psychology and other helping professions, self-care is defined as engaging in behaviours that “maintain and promote physical and emotional well-being” (Myers et al., 2012, p. 56), achieved through the employment of strategies from different “structures of support”, such as the physical (e.g., exercising), social (e.g., making time for family and friends), and emotional structures (e.g., engaging in mindfulness techniques) (Lee & Miller, 2013). In sum, self-care is a series of practices enacted by the individual to prevent negative health outcomes and promote wellbeing.

These disparate strands of theorizing on self-care nevertheless seem to share the common understanding of self-care as a process wherein a rational individual tames unruly emotional and bodily states through the cognitive selection of strategies from abstract and *a priori* categories. In other words, self-care is primarily a goal-oriented, internal process of managing the body through the individual’s manipulation of inert instruments, resources, and environments to achieve desirable affective states. While sensitive to the notion that health is a process rather than an outcome, the body is seen as an object ruled by a dissociated consciousness; and the contexts and structures that support self-care practices are cast as discrete entities existing independently from the individual, rather than inseparable in lived experience (Duff, 2014; Sultan, 2022). In other words, there is little explication of how all these separate elements come together and reconstitute self-care as a relational practice.

As a relational account of self-care, I put forth an approach that emphasizes the embodied and affective practices enacted in the fluid and multi-scalar contexts of the pandemic. In this view, the body is viewed not as a discrete entity separate from the world, as conceptualized in mainstream biomedicine and the social sciences; rather, the body is experienced in terms of its relations and its capacities to feel, affect, and be affected (Deleuze & Guattari, 1987). Following this line of thinking, attention is turned to the confluences of “organic/non-organic confluence of biology, culture, and environment” (Fox, 2011, p. 360), alongside existing and emergent relations of the body unto oneself, others, and the world. Thus, such an approach to self-care also recognizes that the knowledge, practices, and meanings of self-care is a product of people’s engagement with everyday realities (Duff, 2014) and arising from the specific, emplaced interactions of human beings with vibrant nonhuman entities (Andrews & Duff, 2019).

Moreover, the literature on self-care has been implicit in fleshing out spatial realities in concepts such as environmental factors that enable caring for the self, like going outside or being in nature during the pandemic (Fullana et al., 2020). These works imply that self-care is obtained from the environment rather than constitutive of the experience of wellbeing and mental health (Andrews et al., 2014). However, in the context of the social and physical disruptions brought about by the pandemic—especially in the Philippines, where lockdowns are strict and prolonged and vaccination rollout is severely delayed (Mendoza, 2021)—individuals are unable to access practices and places that might have been vital to their self-care. Such disruptions echo an encounter of “throwntogetherness” (Massey, 2005, p. 149) that calls into question new challenges and possibilities of living together in shrunken spaces.

As such, I posit that there is a need to revisit and account for the taken-for-granted nature of the spatiality of self-care. Instead of viewing space as a static and fixed reality—material enclosures where events happen and which are meant to be passively consumed by the human eye (Andrews & Duff, 2019)—spaces in caring for the self can be conceptualized as a creative, dynamic process, as the coming-together of human and non-human elements in the activity of place-making (Duff, 2010), and the context wherein self-care is enacted is seen as “dynamic, always *becoming*” (Sultan, 2022, p. 127). Building on the works of other scholars (Duff, 2014; McLeod, 2017; Sultan, 2022), I similarly put forward a relational account of space to capture the lived realities of self-care amidst the COVID-19 pandemic.

### Applying assemblage thinking in understanding self-care

Weaving all these threads together, I propose to apply assemblage thinking in understanding self-care (Deleuze & Guattari, 1987). Assemblages are “complex constellations of objects, bodies, expressions, qualities, and territories that come together for varying periods of time to ideally create new ways of functioning” (Livesey, 2010, p. 18). As a radical theoretical device, human agency and the individual body within the assemblage are viewed in terms of their relationships and connectivities with other human and nonhuman elements and their capacities to act—to affect and be affected by other bodies, objects, and events (Fox, 2011). While assemblages are provisional in nature, highly dependent on the coming-together of specific elements at particular places, they may endure if associated with habit and repetition; however, they are simultaneously also open in the sense that they may transform into something else (Duff, 2014).

Assemblages may be characterized according to two dimensions: (a) the material or expressive roles of the elements in an assemblage, and (b) the processes by which the assemblage emerges—by which it becomes destabilized and therefore open to change (DeLanda, 2006). Regarding the first point, assemblages consist of material components (e.g., bodies, technologies, spaces) and expressive or discursive components (e.g., identities, affects, desires). However, more than simply enumerating individual components, more attention must be directed towards the capacities and functions within the assemblage, and how the components immanent to it may merge, evolve, or comingle with one another (DeLanda, 2006). By examining the particular confluences of human and material relations, a particular psychic landscape of an individual at a particular moment emerges (Fox & Ward, 2008).

While the application of assemblage thinking seems relatively new in psychological empirical studies, it dovetails alongside new materialist modes of analysis in psychology, where everyday material and embodied practices are recognized as having the capacity to shape subjectivities (Price-Robertson & Duff, 2016) and the representations and meanings of place (DiMasso & Dixon, 2015). Scholars have investigated the ways that places shape psychological phenomena, such as adherence to antiretroviral therapy in HIV-positive men (Robles & Canoy, 2019), experiences of commuter stress (Tenorio et al., 2019), and experiences of wellbeing (McLeod, 2017). Scholars in psychology have also experimented with using material objects in interviews, such as photographs, charts, and other personal mementoes, to demonstrate the power of places and things to provide greater insight into participants’ everyday lived experiences (Craig et al., 2021; Girang et al., 2020; McLeod, 2014; Sheridan & Chamberlain, 2011).

In particular, studies on recovery from mental illness (Duff, 2014, 2016) and drug misuse (Sultan & Duff, 2021) emphasize how recovery, illbeing, and wellbeing are not static but are rather the “emergent capacity to manipulate the affects, signs, spaces, and events of a body’s ‘becoming well’” (Duff, 2014, p. 108). For example, Duff (2014, 2016) demonstrates how recovery is an always-unfinished project of creative resourcing to manage the stigma and symptoms of mental illness, to rebuild one’s life, and to maintain hope for one’s future. In one study, he showed how the coming-together heterogenous human and non-human actors, affects, and forces was vital in shaping atmospheres of recovery (Duff, 2016): Parks and cafes created atmospheres of sociality and belonging; the home and certain arrangements of objects within it created atmospheres of safety and security; and walking encounters with green spaces (e.g., gardens, rivers) created atmospheres of hope and belief. The gathering-together of all these different elements contributed to enhancing the capacities of the recovering body.

Similarly, Sultan (2022) traces the diverse socio-material contexts of drug use and recovery through assembling elements of spaces, practices, and embodiment. She argues that recovery “emerges *in* and *from* drug use” (p. 132) rather than being a phenomenon distinct from it, and that recovery is unique to each body. In her work, she demonstrates how different actors (e.g., the lack of hospital staff, poor living conditions, bodies of others in treatment, funding, etc.) assemble in various ways to render certain spaces as inhabitable or uninhabitable, and how certain material and embodied practices (e.g., obtaining employment, receiving painkillers) shape the rhythms of young people’s ongoing recovery from drug use.

Thus, building on this body of work (Duff, 2014; McLeod, 2017; Sultan, 2022), I suggest a way of understanding self-care as an ongoing practice of assembling encounters with different affects, forces, and practices in spaces in the event of becoming well. Understanding self-care within this emplaced assemblage can flesh out the complex relationship between self and place and show how such relations and arrangements, or cofunctionings (Anderson & McFarlane, 2011), allow us to unpack spatiotemporal processes of healing. Here, the term “cofunctioning” is used to unpack the ways whereby the heterogenous elements of an assemblage come together to “achieve effects and enter relations with other assemblages” (Anderson et al., 2012, p. 176).

### Assembling narratives of self-care

I use a Deleuzian theorizing of narratives to examine the assemblages of self-care (Mazzei & Jackson, 2017; Sermijn et al., 2008). The Deleuzian perspective of the narrative is situated as a critique against the prevailing humanist perspective of the narrative as coherent, linearly structured, and emanating from a single, essential, and knowing subject (Mazzei, 2013). In contrast, narratives within Deleuzian thought are characterized by multiple entryways, multiplicities, and connections and ruptures (Sermijn et al., 2008). There is no one correct entry point that can lead researchers to a single truth about the participant; rather, narratives contain a multiplicity of entryways that present a temporary sketch of the shifting subject (Loots et al., 2017). They are “contexts, rather than just content, or entryways where human and nonhuman entanglements unfold in storytelling” (Robles & Canoy, 2019, p. 3).

Recognizing that assemblages of self-care materialize through embodied, affective, material, and temporal relations, this study asks the question, “What are the self-care practices of university students during the COVID-19 pandemic?” The following analysis will then unpack the different embodied, affective, material, and temporal configurations of self-care as realized in particular spaces in the home, within the context of the participants’ everyday lives during a global pandemic.

## Materials and Methods

### Recruitment, sampling, and study population

This screening, recruitment, and interview protocol was implemented after ethical clearance was obtained from the University Research Ethics Office of the Ateneo de Manila University, ID No. AdMUREC_20_057. A purposive sampling approach was used to recruit participants for the study. Due to the pandemic lockdown restrictions, all participants were recruited online via advertising on the social media pages of private and public university student organizations. Along with the call for participants, a short survey was appended containing the Perceived Stress Scale (PSS-10) (c), questions regarding the potential participant’s engagement in self-care practices, and their willingness to be contacted for an interview after the completion of the survey. The PSS-10 measures the degree to which situations in one’s life are appraised as stressful and is considered a psychometrically sound global measure of perceived stress in normative samples of college students (Roberti et al., 2006). This scale was included to provide information about a potential participant’s state of mental health, which allowed me to be more sensitive to the psycho-emotional context of those who decided to proceed with the interview. Additionally, those with high levels of stress according to the PSS-10 were to be excluded from the study to avoid potentially triggering participants during the interviews.

A total of 25 participants between the ages of 18 and 21 answered the screening survey over the month and a half that it was advertised online. Majority of those who answered the survey identified as female (88%), were currently studying in a private university (76%), and were residing in Metro Manila (68%) at the time of the survey. Metro Manila, is a densely urban region with the highest number of COVID-19 cases in the country (World Health Organization, 2020). Of the 25 participants, seven declined to be contacted for an interview, so they were automatically excluded from the study sample.

The remaining participants were then screened for their engagement in self-care practices by indicating their response to the question, “Do you identify as engaging in self-care practices, however you define them?” In line with the ontological and epistemological assumptions of the study, I did not impose any pre-existing notion of “self-care” on the participants; rather I allowed them to self-identify according to their personal resonance with the term. Four participants indicated a negative response to this question, and so were excluded from the study.

Following this inclusion and exclusion criteria, a total of 14 participants were eligible to be contacted for an interview via the email they had provided in the survey. Three of the 14 participants declined to proceed to the interview due to not having a stable enough internet connection, while the other 11 consented to be interviewed following the receipt of the email. Upon their indication of verbal consent, they were given the Informed Consent Form to review and were invited to an orientation session regarding the study prior to the interview proper, which will be described in more detail in the next section.

Thus, a total of 11 participants were interviewed (90% female, 64% from a private university). All participants were living in the National Capital Region at the time of the interview and were living with their families of origin. The interviews were conducted within January to February 2021 to coincide with the semestral break of the students from online classes. Additionally, the principle of information power (Malterud et al., 2016) was used as a guiding principle in determining the final sample size for this study. Given certain practical considerations, the relatively narrow aims of the study, the strength of the interview dialogue, and an analysis that includes an idiographic focus on narratives, a smaller sample size—between six to 12 interviews—is deemed sufficient (Guest et al., 2006; Malterud et al., 2016).

Despite initial efforts to capture a more diverse gender and socioeconomic profile by recruiting an equal number of male and female participants and equal number of participants from private and public universities, the sample criteria were adjusted considering the practical realities of the research process (Braun & Clarke, 2019b). For instance, one limitation is that because of the pandemic, participants could only be reached through online advertising. Those who were able to see and respond to the online survey in the first place also tended to be from families of higher income levels with better access to technological resources (Robinson, 2014). This is especially true in the Philippines, where internet speeds are among the slowest and most expensive in the world, particularly in rural areas (Laforga, 2020). This could be one reason why there were more participants in this study from private universities than from the public universities.

Another limitation of any study requiring interviews is the presence of a self-selection bias (Costigan & Cox, 2001), wherein those who consent to be interviewed are generally more open to intimate self-disclosure than the general sample universe (Robinson, 2014). In this study, majority of the participants who signed up for the interview identified as female, and research has shown that females have a higher tendency towards emotional self-disclosure than males (Sultan & Chaudry, 2008). This self-selection and female bias is not entirely possible to circumvent, since voluntary participation is necessary for sound ethical practice (Robinson, 2014). However, it is possible to acknowledge this limitation and how it shapes the interpretation of the findings (Braun & Clarke, 2019b).

### Data collection procedure

The following data collection procedure is a methodological experiment inspired by the approach of “orientating to assembling” as outlined by McLeod (2014). In using the theoretical device of the assemblage to inform the data collection process, McLeod (2014) recommends to (a) make materials central and (b) include nonhuman connectivity during the research process, which are crucial in questioning the centrality of human interactions in the overall research project. Introducing nonhuman objects—particularly photographs—during interviews allowed me to direct my attention to the entanglements of human and nonhuman interactions. For this study, I adopted the autodriven photo-elicitation interview as the primary mode of data collection, in which the participants themselves provide the photographs for the interview in relation to the research topic (Clark, 1999). This allows them to take a more active role in the research study and provides a quick means of establishing rapport (Clark, 1999). Photographs also serve as symbolic forms of representation for nonhuman and human elements involved in the participants’ self-care practices, giving expression to what might have remained unsaid in a verbal interview (Harper, 2002).

Guided by these orientations, the stages used in this study were as follows: In the first stage, participants who consented to being interviewed were invited to an orientation session regarding the study, especially in the taking of photographs. In the intermediary stage, participants were given three to five days to take the photographs and to email them back to me before the scheduled interview. Finally, the last stage consisted of the photo-elicitation interview-one-on-one online synchronous interviews where participants walked me through the photographs they took and where I asked them more questions regarding the photographs. Each of these is described in further detail below.

#### Stage 1: orientation session

Once participants gave verbal consent via email to being interviewed, they were invited to a brief orientation session via audio or video call on Google Meet that lasted about 20–30 minutes. The purpose of the orientation session was to establish rapport with the participants and to orient them regarding the specifics of the study. During the orientation, participants were (a) introduced to the context, purpose, and process of the study; (b) briefed regarding the Informed Consent Form; and (c) oriented to the guidelines in taking photographs on their phone regarding their self-care (see Box 1 for more details). Participants were also briefed regarding the ethical measures to be taken to protect their privacy in the photographs, such as blurring certain aspects of the photographs that might provide environmental cues to their location or identity in the final paper.

**Table:** 

**Box 1**: General Instructions for Taking Photographs Take around 8–10 photos with your phone or whatever camera you have available of the spaces, objects, experiences, or even people that have been meaningful to your self-care since the start of the lockdown until now. It can be anything, even things that might seem so ordinary or mundane or “not Instagrammable” (e.g., your plants, a favourite spot in your house or neighbourhood, a certain view—it’s up to you!). You can be as creative with your photos as you want. Think of it like your personal project, a unique collage of what self-care is to you. But also, try not to overthink it—try not to pressure yourself to come up with something wildly unique or special or photogenic. Just choose whatever comes to mind and whatever you feel is really you. You’ll have around 3 days to take and choose the photos. You can send your final set of photos to my email. During the interview, you can show me the photos again, and you can tell me more about what they mean to you.

#### Intermediary stage

During the intermediary stage, participants took photos of spaces, objects, or experiences in their home or their immediate environment that they considered as vital to their self-care practice and mental health, especially in the context of the pandemic. Given the digital nature of the photographs taken, some took the liberty to be as creative with the presentation of the photographs as they liked, compiling them into a PowerPoint presentation or giving the photographs filters and labels according to their own preferences.

#### Stage 2: photo-elicitation interview

Due to the limitations on mobility during the pandemic, all interviews were done online and synchronously through Google Meet and lasted approximately 40 minutes to an hour. Online synchronous one-on-one interviews entailed that both the participant and I were online simultaneously to engage in a real time conversation, which offered the safety, ease of access, and convenience while preserving the face-to-face experience (Jowett et al., 2011). This also preserved a sense of private space for both the participant and I as we encountered each other in the safety of our own locations (Hanna, 2012).

Right before the photo-elicitation interview, some formalities and ground rules were discussed for the psychological safety of the participants. First, participants were reminded of their rights and the nature of their participation based on the Informed Consent Form. They were assured that their information will remain confidential. Second, since we were not in the same physical space, privacy was ensured by asking the participant if they were alone in a space where they could remain undisturbed for about an hour. Participants were also informed when the recording would begin and were allowed to adjust their preference for audio or video call depending on how comfortable they were with those options. They were then reminded that they could refuse to answer questions they do not wish to answer for any reason and were also briefed regarding what might be done should a line of questioning be triggering to them (e.g., stopping the recording and doing a breathing exercise together). However, the latter protocol did not need to be activated during any of the interviews, as none of the participants were triggered during the process.

Once the formalities were accomplished, I began the interview proper by asking participants to tell me about the photos they took through the screen-share function on Google Meet. Following a radical approach to narratives (Sermijn et al., 2008), participants were told to start wherever they wished and with whatever photograph they were drawn to. They were all given the following opening prompt: “This interview will be free flowing, so I’ll just ask you to begin wherever you want to begin. How have you been taking care of yourself? You can choose any photo to start with, or you can feel free to start anywhere.”

My position as a researcher shifted dynamically over the course of the interview. During the initial sharing of the photographs, I found myself positioned as a witness to the participants’ stories, allowing myself to be affected by the photographs (McLeod, 2014). At other points, I took a more active position in probing for the participants’ affective relations with the objects, spaces, and people in the photographs, and how these had shaped their self-care practices. Some questions that were used to probe were “What feelings are coming up for you in these photos?”, “How did you use these spaces/objects/experiences in your self-care?”, and “How have these facilitated your self-care practices?”

Interestingly, the share-screen function also allowed participants greater freedom in informally showing me other photographs, apps, and windows on their screen that elaborated on what they had already captured and revealed other aspects of their self-care over the course of the interview. The materiality of the digital platform thus allowed the interview to branch out in unexpected directions (Michael, 2012), allowing for greater leverage in terms of interpretation and insight.

### Data analysis procedure

Each interview was recorded by the researcher and transcribed afterwards. To protect their anonymity, all participants were given pseudonyms, and any identifying information in photographs were blurred. In the final analysis, the interview with the male participant was excluded to sharpen the analytic focus of the study and to allow for the better contextualization of the results.

Following this, I drew from McLeod’s (2014) iterative approach of making maps, crafting assemblages, and identifying their cofunctionings to analyse the data. Additionally, consistent with other reflexive and interpretative approaches to qualitative methods (e.g., Braun & Clarke, 2019a), I view data analysis as an embedded, context-bound, and fluid process of meaning-making and knowledge construction instead of the excavation of *a priori* meaning through the systematic identification of codes (Jackson, 2013). Within this view, researcher subjectivity is viewed not as a liability but rather as a valuable resource in enriching the overall research process and outputs (Clarke, 2005; Gough & Madill, 2012).

**Figure 1.**
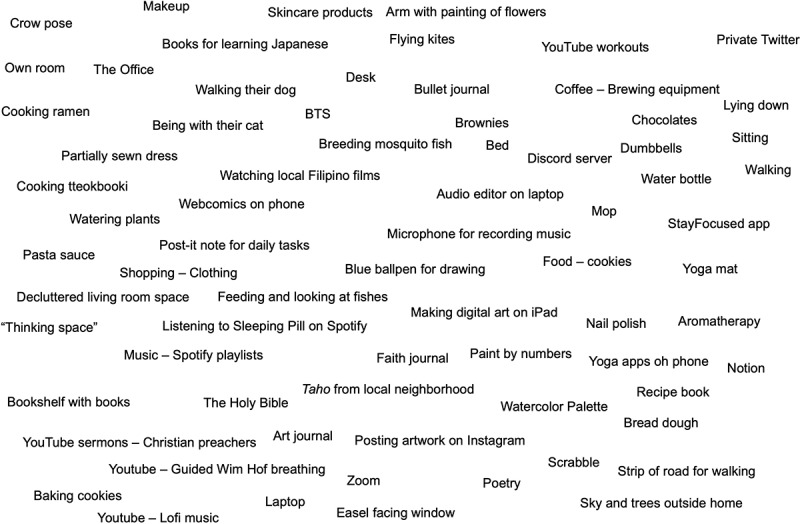
Map-making, part 1.

During the initial stage of analysis, I familiarized myself with the transcripts and engaged in the iterative process of memoing and map-making, which involved working at the surface with transcripts and photographs (McLeod, 2014). Map-making emphasizes the temporally emergent connectivities between human and nonhuman elements in particular spaces and places (Deleuze & Guattari, 1987) rather than simply the discursive content of the interviews. I used a modified version of Clarke’s (2005) situational mapping in drafting the initial version of the human and nonhuman elements involved in self-care (see Figure 1). This stage of the map-making process is intentionally messy—it is intended to be analytic exercise that is accessible and easily manipulated by the researcher (Clarke, 2005). Some questions using assemblage thinking that guided the analysis were: (a) Which bodies are involved in self-care? (b) What material elements—objects, spaces, places, etc.—are involved? (c) What affective states are evoked by the photographs in the narratives?

**Figure 2.**
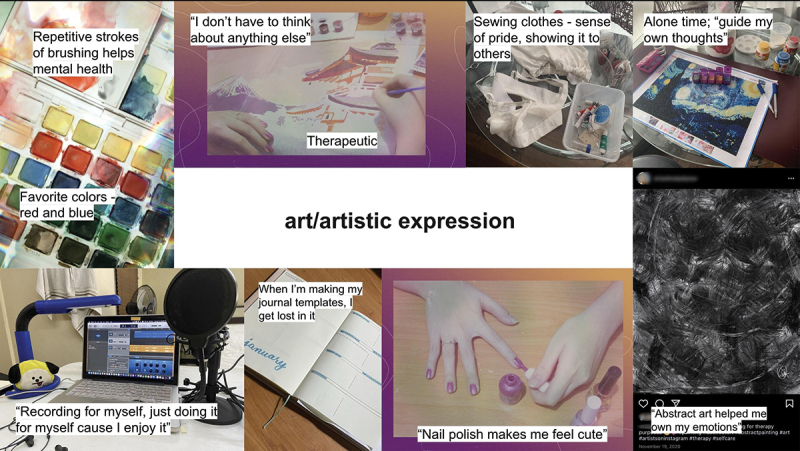
Map-making, part 2.

In the next stage of analysis, I attuned to how practices of self-care were reinstituted within the home and how these co-mingled to form assemblages. As new connections and ruptures were enabled between the elements of the drafted map, I then transferred to working on a digital space to facilitate the re-arrangement of photographs and the extracts into broader collages (see Figure 2). With this, questions guiding the interpretative identification of assemblages and cofunctionings included: (a) How are the bodies affected, and are affected by, the other elements? (b) How do they demonstrate the complex interplay of elements in relation to the enabling and limiting capacities of the body? (c) How do spaces and objects enable the capacities of participants to care for themselves? (See Box 2 for an example of the memoing process.) Crafting assemblages is therefore not simply about identifying their individual components or properties, but about tracing the complex interactions between these components and developing an attentiveness to the whole (Sultan, 2022).

**Table:** 

**Box 2**: Sample Memo for Figure 2 Provisional name for assemblage: art/artistic expression. The elements of brush-paint-canvas-numbers, the “repetitive” nature of painting, facilitates affective states of wellbeing, particularly a state where only the material exists and thoughts/human chatter in the mind ceases: “I don’t have to think about anything else”; in the case of crafting templates for her journal: “I get lost in it, everything around me just disappears.” One labels this as *focus*. In another one, jewel painting: The beads-canvas-numbers assemblage—an opportunity for alone time to “guide my own thoughts”, just as the beads are being guided on canvas. Contrasted to the alone time on her phone—the phone *limits* self-care because her thoughts are always influenced by others (social media, messaging apps). Related to this is another shifting form of this assemblage—digital art: tablet-pen-app. The function: “Helped me own my emotions.” Another form: paint-brush-body, or polish on nails—“Makes me feel cute.” Perhaps facilitates confidence.

### Validity and reflexivity

Throughout the entire data collection and analysis process, several strategies outlined by Yardley (2015) were employed to enhance validity. First, sensitivity to context was observed through adopting a “reflexive scientific attitude” (Gough & Madill, 2012, p. 375) during the interviews with participants, recognizing that my own position as a researcher also invariably shaped the findings of the study. However, I also guarded against the assumption that I would share the same language and meanings as the participants in viewing self-care practices by keeping a personal research diary, wherein I examined how my assumptions, expectations, and values influenced how I interacted with participants and interpreted their narratives (Finlay, 1998). I also did not impose any preconceived notions of self-care on the participants, whether my own or from the literature (Yardley, 2015); rather, I recognized that these patterns and meanings would be co-constructed through the human and nonhuman interactions during the interview and analysis process (McLeod, 2014). Second, in line with the principle of coherence and transparency, I kept a detailed paper trail of maps, memos, and reflections throughout the research process to record the evolving analysis and all decisions made at each phase of the study. Finally, the principle of commitment and rigour was observed through an in-depth engagement with the topic during the analysis and by substantiating findings with rich descriptions from the participants (Yardley, 2015).

## Results

### Work-home assemblage in the context of the pandemic

I situate the process of embodying lived practices of caring for the self during the pandemic within the makeshift work-home assemblage. In the context of pandemic, the inflow of the coronavirus into public airspaces between human bodies corresponded to an outflow of human bodies and activities from the public sphere, creating multiple ruptures in the participants’ lives. The material public health measures in response to the virus, such as the continuous enclosures of public places and the “stay at home” orders of the Philippine government, had affective consequences on all participants. For example, prior to sharing their photographs, about half of the participants experienced negative affective states in response to the lockdowns:

At the start of the pandemic, the main problem for me was that suddenly I had so much free time. There were just so many things that I could do with that free time, and the sheer number of possibilities was paralyzing. So I ended up waking up and then not doing anything at all, or waking up and then wasting a lot of time on Twitter. (Riley)

I didn’t really expect the pandemic to happen. Everyone was so shookt [sic] about everything going on and no one knew exactly what was happening, so at the start, I was so confused with my life … Every day felt like [I had] nothing to look forward to, because there was that uncertainty [of] what will even happen in the next few months.(Grace)

In the extracts above, the sudden inflow of unbound time within the home produced the affects of paralysis, confusion, and helplessness in participants, diminishing their capacities to act and be acted upon by their environments. For participants like Riley, inherent in the excess of unbound time is also an excess of possibilities—a proliferation of imagined temporal projections into the future—which in turn produced a corresponding “paralysis”, an immobilization of her body. On the other hand, Grace felt like she had “nothing to look forward to” during the pandemic because of the “uncertainty” of what would happen. Initially, during the first months of the pandemic in 2020, her anchor was the resumption of onsite face-to-face classes. However, when the first and second semesters remained online for the next school year, it made her feel “so much worse” because it made her realize that “things won’t go back to normal”. In other words, participants like Riley and Grace felt a sense of being suspended in time, unmoored from the spaces and embodied practices which guided their day-to-day rhythms (e.g., primarily going to face-to-face classes and going out with their friends and family members).

In their narratives, half of the participants also constructed “turning points” where they realized that something must be done to change their situations. According to Grace:

Then little by little, I was like, “You gotta live with this. You can’t do anything. This is beyond your control. You just gotta find a way to get through this. Like that’s all you can because you’re at home”. (Grace)

It was also this point in the interview that participants showed me their photographs on the objects and spaces that constituted their practices of caring for the self. Thus, it was the presence of this pervasive uncertainty and the absence of routines enacted in spaces outside of the home that new ways of caring for the self were assembled.

In the section below, I unpack the emergence of the work-home assemblage in the context of the pandemic, and then elaborate upon its different cofunctionings. The work-home assemblage is composed of spaces, bodies, and objects within the home in specific spatial and temporal arrangements that lend it a provisional identity (Duff, 2014). In this case, the work-home assemblage is first delineated by an outflow of unbound time and undifferentiated space and an inflow of (a) boundary-making and place-making practices and (b) the creation and re-creation of time spaces within the home. Instead of being a monolithic space representative of the private sphere, the home is broken down into different pockets of space where previously public activities, such as going to classes, the gym, or a café, are reinstituted.

#### Boundary-making and place-making practices of work-home

More than half of the participants emphasized the importance of creating boundaries between schoolwork and home as part of their self-care. Ariadne depicted her boundaries this way:

I found it important to have a certain boundary between work and rest at home. Like, I have a certain study desk and I made a rule for myself never to study in my bed or study lying down.

**Figure 3.**
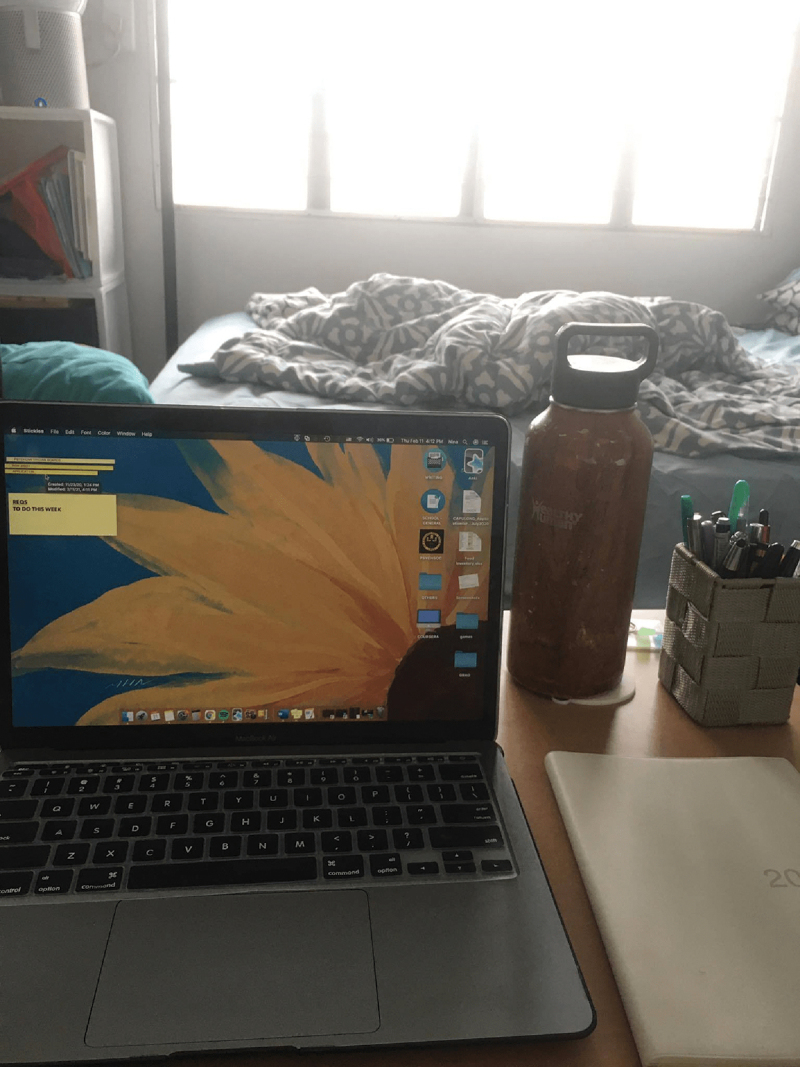
Boundaries between work and rest.

Figure 3 shows Ariadne’s workspace in the foreground—her desk, laptop, and planner—and her bed in the background. While her desk and bed co-exist in the same room, Ariadne nevertheless considered this a separation of space. This demonstrates that boundary-making during the pandemic does not necessarily consist of moving to different enclosed areas within the home, but rather shows that *objects have the capacity to create space and generate different affective relations within space*. Here, Ariadne’s desk created the micro-space of work and her bed the micro-space of home. Furthermore, this reveals that certain bodily positions in space also create transitions from one micro-space and affective state to another. For example, Ariadne never studied on her bed, and she avoided lying down while she was working. In this way, her body signalled to her the transitions between the different affective states of work and rest. Thus, part of the practice of caring for the self also involves establishing certain rules bound to the materiality of the micro-spaces recreated.

Aside from the practice of boundary-making, another practice that a few participants named as part of their self-care is the process of place-making (Duff, 2012)—particularly, in recreating their favourite places pre-pandemic in the space of the home. Here is what Ariadne called her “thinking space”:

I like to replicate certain environments, especially in the context of the pandemic. There’s a certain space in this restaurant or café I like to pick to people-watch because I don’t like being a in an enclosed space, I like being a place where it’s busy but it’s not chaotic. So I like this space because it’s a balcony where I can watch people, and in a way, feel connected with the world.

In the extract above, Ariadne found that she had to make do with the pre-existing spatial and material configurations of her home to recreate the cafés she used to afford her pre-pandemic—places where she used to do her best thinking through reading and writing. With a little experimentation (i.e., with the addition of a chair from the living room and the addition of a few more plants), Ariadne found that the balcony was able to simulate a similar atmosphere of openness and sociality of cafés. Here, Ariadne’s practice of place-making demonstrates how individual elements in the assemblage (balcony-chair-plants-unbound air-space) constitute the whole but at the same time, exceed the whole. Through a process of bodily immersion in one’s practical home environment and the remembering of a pre-pandemic space, the function of the balcony is transformed into a thinking space—café.

#### Creation and re-creation of time spaces

**Figure 4.**
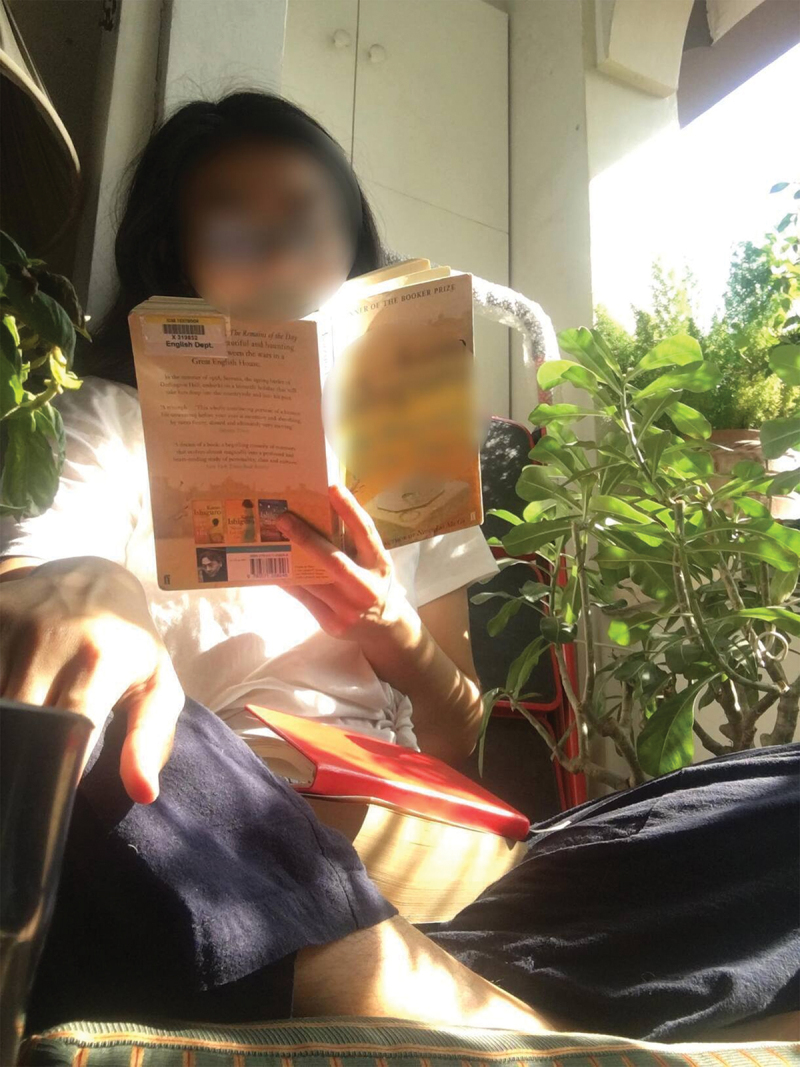
Dailies (left); Master schedule (right).

With the loss of public spaces for bodies to navigate, there was also a disruption in the temporal rhythm of bodies, which participants sought to re-establish in their self-care practices. One way that participants expressed doing this is through building a routine. For example, Riley folded the unbound time of the pandemic into physical spaces and embodied practices through a sticky note posted on the corkboard beside her bed (Figure 4, Left).

She called these sticky notes her “dailies”, a list of activities she did upon waking. She explained, “My routine ensures that I do things that benefit me upon waking. It prevents me from lying in bed for too long.” In this way, the sticky note acted upon her body by re-establishing a new temporal rhythm for her mornings.

**Figure 5.**
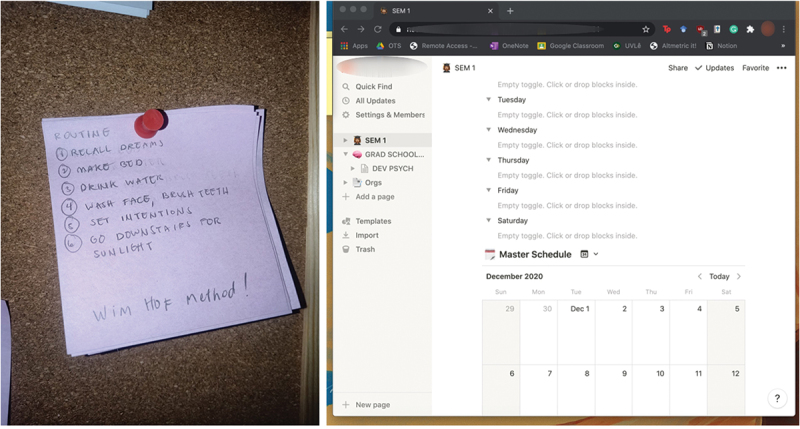
Thinking space.

On the other hand, for Ariadne, her time spaces are produced through objects (see Figure 5, Right) and through a conversation with her body. She said:

What I do is I have a certain schedule, like from 10 to 5, and it really helped me. By doing this your body gets used to working at this time and your body gets used to resting at this time.

For Ariadne, the production of this time space occurred through both formal and informal means: First, through an attunement to what she called her body’s “optimal time”, she constructed the time interval of 10 AM to 5 PM as the time space for her work, marked by a clear purpose and duration. The platform Notion also aided her in organizing this interval into smaller time spaces for specific tasks. Thus, this time space emerged from a meshwork of virtual classes, Ariadne’s own embodied experience of her temporal rhythms, and digital platforms that allow for the visual parcelling of time into smaller chunks.

### Cofunctionings of the work-home assemblage in caring for the self

In the following section, I elaborate upon the different cofunctionings of the work-home assemblage, delineating how the collaboration of human and nonhuman elements enable practices of self-care and give rise to certain affective experiences. Here, I use the word “affect” to mean the energies and intensities between human bodies and material objects, experienced by human actors as feeling-states, and a “willingness and capacity to act” in response to these states (Duff, 2010, p. 882).

#### Caring for the self as fostering different relationalities with the self

In this section, I unpack how the work-home assemblage of self-care functioned to foster different relationalities with the self, enacted through collaboration with different spaces and objects. First, it functioned through extending the capacities of the body to act through the diffusion of felt intensities. Second, this assemblage also transformed one’s relationality with one’s emotions by the aesthetic re-embodiment of messy intensities. Third, it enabled the healing past hurts and wounding, rendering it capable of living through the present and future with hope. Finally, concurrent with all these cofunctionings is that it allowed participants to attune to the present embodied self over time.

*Diffusing negative felt intensities*. Many scholars have documented the physical and psychological effects of physical self-care practices, such as regular exercise and proper diet, on the human body. However, since places of wellness such as the gym, yoga studio, and the green university campus have closed, participants had to reconstitute pockets of wellness within the home. The objects they chose for physical wellness not only recreate these everyday micro-spaces, but also extend the capacity of their bodies to act by diffusing negative felt intensities.

**Figure 6.**
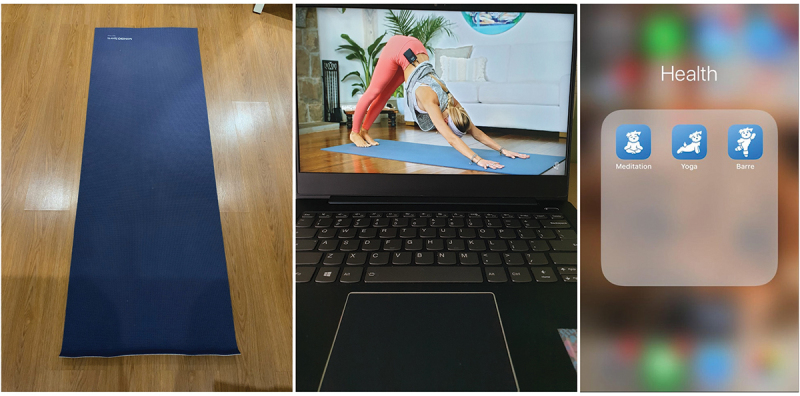
Yoga.

For example, a third of the participants included photos of their yoga mats and an app or a screencap of a YouTube video (see Figure 6), which recreated the space of the “yoga studio”. They mentioned that they only took up a physical movement practice during the pandemic because the nature of online classes obliged them to be in a seated position all day. As Kate put it:

Yoga is so important to me because there was a point in the pandemic where I realized that I had a lot of tension in my body. Like I describe as my energy being pent-up in my head and not being utilized by the rest of my body. So when I do yoga and I feel like my muscles getting stretched and I do all the poses, it kinda helps to ground my energy. I know it’s benefiting my body and it’s also alleviating my anxiety.

In the extract above, Kate valued yoga not only for the practice itself, but also for what it can do for her body—the yoga mat-laptop-app-yoga poses meshwork enabled for the diffusion of felt anxieties. Moreover, this extract demonstrates that this collaborative meshwork of human and nonhuman creates a complex affective space for Kate: A seated bodily position created a sense of tension, stress, and anxiety; while the process of moving through a yoga flow created the affective experience of calm, grounding, and relaxation from stress and anxiety.

As Kate learned to synchronize different sensations that flow through her body-in-space, performing yoga as an everyday embodied practice also helped her manage bodily tensions over time:

I have a generally fast pace especially when I’m doing deliverables so I need to deliberately inject things that will slow my pace, because when my momentum builds as I’m studying or as I’m doing orgwork, the tension in my body kinda builds up and that’s where my stress and anxiety come in.

In Kate’s experience, stress and anxiety emerged not only from the nature of the work, but also from the build-up of the work *over time*—in this case, the “fast-paced” nature of the deliverables of school and extra-curricular work. While synchronic or aligned flow is desirable through successful management of affective states, an embodied space of temporal discontinuity—breaking off from fast (societal) rhythms by maintaining a slow-paced body—may need to be reconstituted through active injecting of self-care activities. Thus, extending the physical self in this context ascertains multiple time spaces of embodied care.

**Figure 7.**
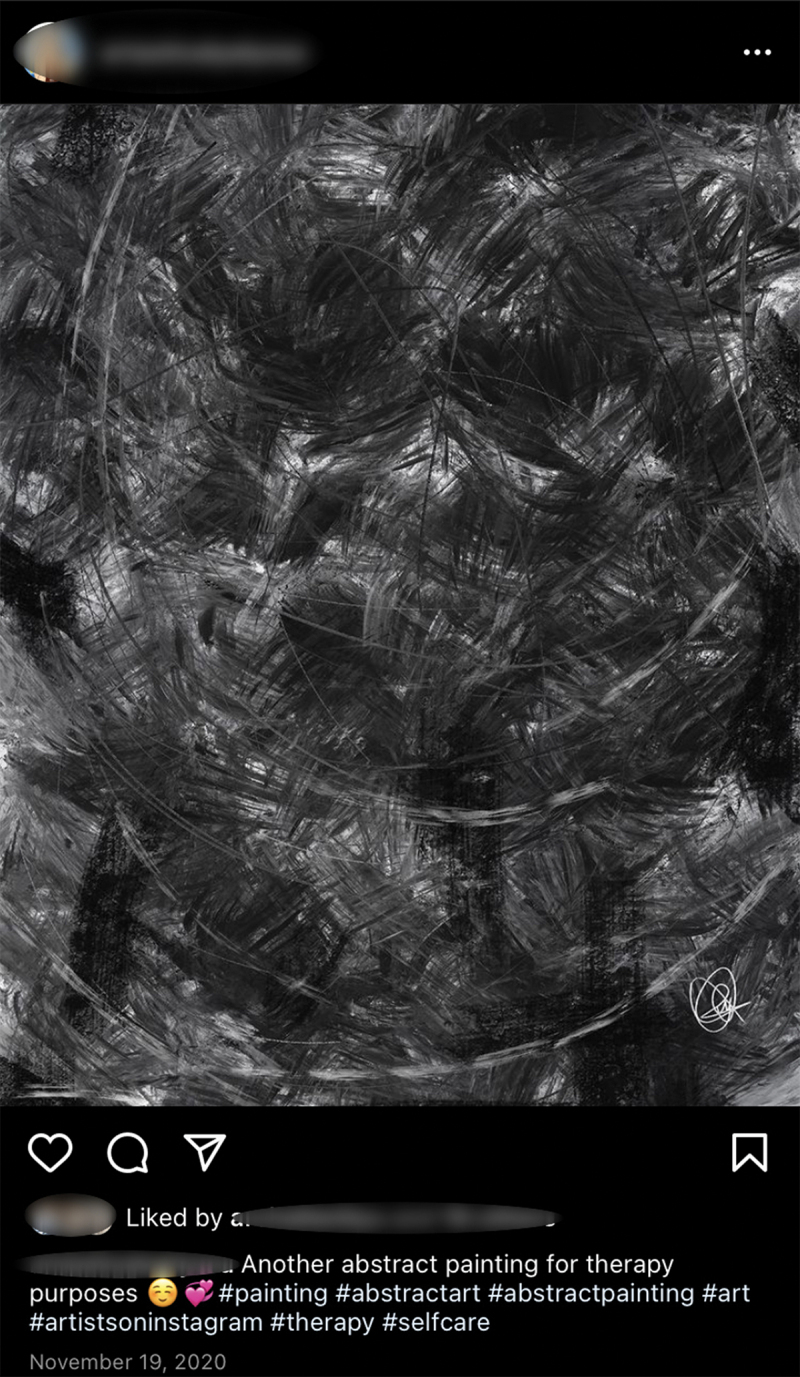
Abstract painting.

*Re-embodying felt intensities*. Another meshwork of objects related to the event of self-care in a third of participants related to creative or expressive practices, which, instead of simply diffusing felt intensities, re-embodies them. Zoey spoke of this process of re-embodying affect through creating her own paintings (see Figure 7). She said:

What I did during quarantine was that I started to allow myself to feel my emotions, and I don’t always talk to my friends about it, so I try to find other outlets. And what I usually do is drawing. Like if I felt negative emotions that I couldn’t express I would try to draw it … I just really started trying to own my emotions.

In the extract above, Zoey described the piece as an embodiment of her emotions—an extension of her own body in space—when discursive practices like talking to her friends were unavailable to her. Painting served to materialize emotions through the *embodied transformation* of felt intensities. Here, the painting was not only a blank canvas upon which Zoey inflicted her emotions; rather, this piece of art also acted upon her and myself as the researcher during the interview as we viewed and discussed this piece of art together, knitting all of us together in a collective body of sense-making and care.

This analysis also follows from the principle of Deleuzian narratives as having connections and ruptures (Sermijn et al., 2008). With this, connections between events do not exist in pure form within the subject, but rather are tenuous and situated within a certain context (Sultan & Duff, 2021). For example, when I asked Zoey to walk me through what emotion this “meant” to her, she stuttered and laughed, saying, “I don’t know how to explain this, I’ve never had to do it before.” Given my prompt, however, Zoey said that it did not represent any one emotion but rather the very *messiness* of her emotions. Entangled, assembled, and enabled by the relationality of interviewer-interviewee-artwork, she extrapolated that even in the darkness of the pandemic, she realized that “grey areas” existed, which made her reflect on how “life is messy but also very beautiful”. Additionally, while the process of drawing created an affective space considered as “therapeutic” and “calming” for Zoey, her artwork itself did not follow a linear progression from negative affect to positive affect; rather, it depicted the co-existence of *both* within the space of the canvas, allowing Zoey to physically view, tolerate, and transform her relationship to the intensity of negative emotions, thus reassembling and transforming one’s relationality with the self.

*Healing temporal felt intensities*. For some participants, the practices of caring for the self also enabled them to develop different ways of relating with their past selves and their future selves. This follows from the narrative principle of multiplicity, which acknowledges that the subject has multiple stories that cannot be reduced to a whole; rather, the participant is multi-voiced and always shifting (Sermijn et al., 2008). Thus, for participants, healing pertains to the process of recognizing and accepting temporally discordant selves through the inscription of these multiple selves on the body (e.g., Dennis, 2020).

**Figure 8.**
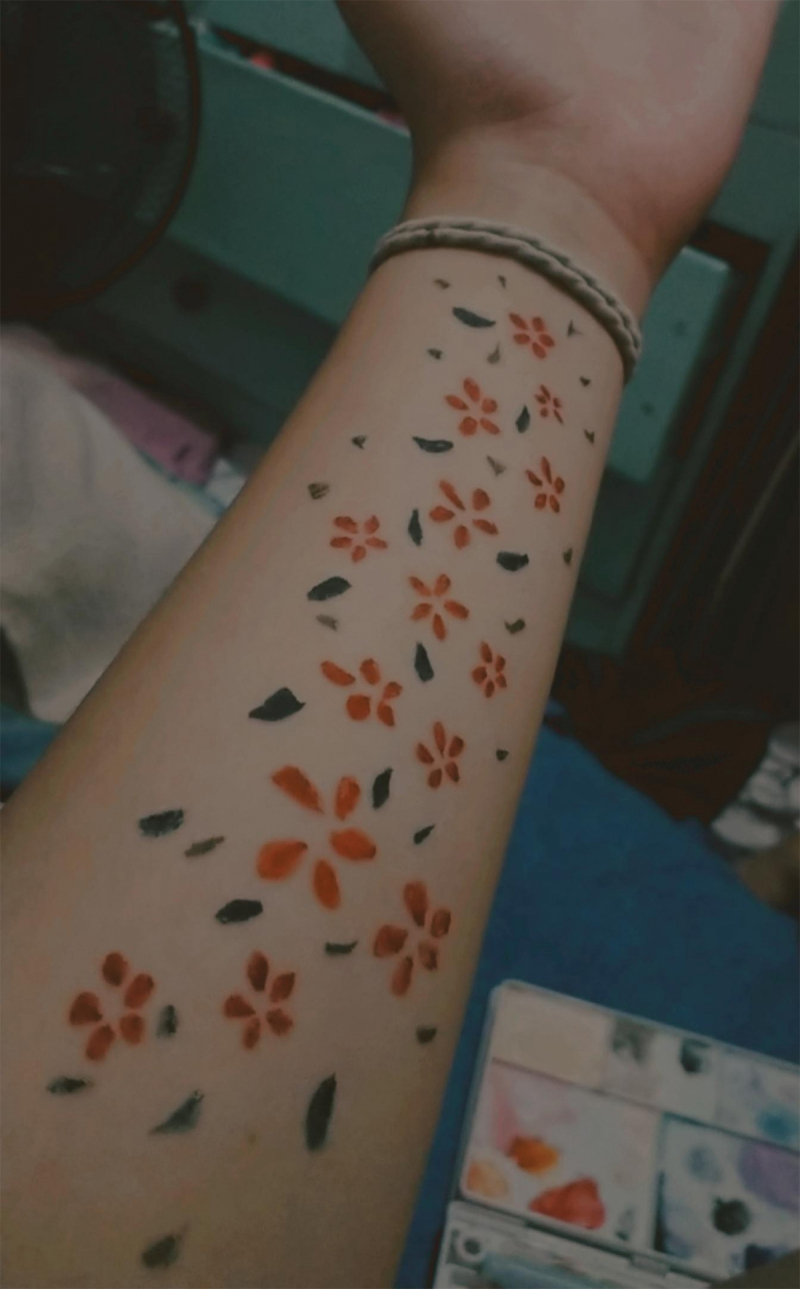
Painted flowers on skin.

One participant, Lila, will be used as the exemplar in this case. Lila disclosed that while she had never been formally diagnosed with a mental health condition, she struggled with self-harming tendencies in her teenage years. She was already “clean of self-harm” for six years at the time of the interview. However, one time during the pandemic, she’d accidentally pressed a pen too hard on her skin while she was drawing on it, creating a small scratch. Not wanting this to become a pattern of harming herself, she resolved to set “boundaries” around this behaviour by replacing the pen with a brush (see Figure 8). She said:

On a really bad day [when] I’m really frustrated, I would paint on my skin because you know how people say that it feels like their skin is crawling? Yeah and painting on my skins helps me because of the different sensation of the brush. If I do something with my skin, I would remember what what’s the truth, what’s the reality, and what’s actually happened. Then I leave [the paintings] overnight and then when I wake up it’s like a reminder that I did good and I got through the night without doing anything bad.

In the extract above, the flowers on her skin simultaneously embodied multiple temporally discordant selves—the past, present, and future selves are materially engraved in the body as *all real* or fully embodied. First, this practice served to facilitate a different relationship with her past self through embodied storytelling. The past self was embodied for her through the feeling of her “skin crawling”, which was a trigger for self-harming behaviours. For Lila, the act of moving the brush over the skin helped soothe the crawling by providing a different tactile sensation on her body. Here the body, engraved red flowers, and green leaves served as a material reminder to Lila’s present self that she “did good” and that she “got through the night without doing anything bad.” This produced the affect of relief and safety, which concretely signalled to her that she can entrust herself with the safety of her own body. The practice of painting on her skin was thus an act of reclaiming agency of the past wounded body: When previously, the self-inflicted wounds experienced by a past self slowly rendered her body uninhabitable, through the act of painting, Lila transformed and constantly affirmed her will to live and to fully inhabit her own body.

This assemblage also reached forward into Lila’s future. When asked about the colours she chose and why flowers and leaves, Lila mentioned that the colours “remind me of hope”: “When I paint on my skin, I just remember the good parts of my life and how enjoyable it is and how I want to paint more permanent paintings.” In this utterance, Lila projected her body into a livable future where she can continue doing what she loves. Thus, this cofunctioning created the affective encounter of hope. Assembling self-care practices not only diffuses multiple intensities by extending the physical self and re-embodying them, but also enables a deep healing of a liveable self by moving forward through life with hope.

*Attuning to a present self*. Finally, co-occuring with diffusing negative intensities, re-embodying felt intensities, and healing temporal felt intensities is the cofunctioning of *attuning to the present self*. Within this cofunctioning, participants talked about how assembling different self-care practices allowed them to listen more deeply to their bodies and address their unique needs. As Kate put it:

I had a very superficial definition of self-care because what I saw constituted self-care on social media was skincare routines. But then in the pandemic that’s when I realized that like self-care really is about genuinely about taking care of yourself and addressing your personal needs, and it will vary from person to person.

In the extract above, Kate described how self-care is not about the adoption of the popular, pre-existing self-care practices of others, but rather about “really” and “genuinely” addressing one’s personal needs. There was a recognition that practices of self-care vary from person to person because each “self” is different from the “self” of others. Thus, caring for the self involves an attentiveness to what the “self” is experiencing and what objects and spaces exist within one’s environment to facilitate those caring encounters.

Aside from the recognition of a self as different from others, there was also the recognition that this self that changes over time. What is considered the “self” is not static but rather dynamic and in flux. According to Maddie:

Over this pandemic there were other activities I’ve stopped doing because it’s something I didn’t enjoy and I was just doing it because I thought it was self-care. So I guess self-care is giving myself the choice on what I want to do and need to do.

For Maddie, self-care was an ongoing choice to do things that were aligned with her needs on a day-to-day basis, particularly the choice to do self-care differently than from her past self, knowing that she wasn’t bound to repeat her previous choices just because she “thought it was self-care.” This choice was something she continually gave to herself. The word “give” here implies a relationality with the self, and part of attending to the self is the capacity to give to oneself.

Other participants like Zoey and Ariadne also discussed this dynamic of giving to the self and developing a relationality with the self, particularly in the context of self-care being considered as “selfish” in popular discourse. They said:

When you’re doing self-care, it’s like you have to be attentive to yourself. I know like some people think it’s like selfish, but I honestly don’t feel that way. For me it’s trying to learn how to appreciate myself in my own company. (Zoey)

Self-care is not really selfish … It isn’t just about the self because you don’t just work for yourself. If you do practice self-care, it makes you healthier, and it also affects the people in your environment and the work that you do. (Ariadne)

These participants grappled with the moral accusation levied against self-care as being “selfish”—that they are taking the time and attentional resources that should be reserved for others and instead hoarding it for the self. However, in their experience, caring for the self was not about taking from others, but rather giving to one’s own self, and cultivating a nourishing relationship with the self and its different configurations over time. The moral ambiguity of caring for the self was resolved when this relationality with the self is seen as equally valid as one’s relationality with others.

### Caring for the self as copresencing with human and nonhuman others

With the outflow of human beings from public spaces is a corresponding alienation from the participants’ networks of peer support. Given this, participants collaborated with different physical objects and digital platforms, experimenting with different ways to establish spaces of care with their peers. In these assembled spaces, there was a renewed appreciation for the presence of life itself in their respective spaces, namely, pets and plants. Thus, in this section, I unpack two broad ways of copresencing: (1) copresencing with human others, and (2) copresencing with nonhuman others.

*Copresencing with human others*. In the absence of the physical human bodies of their peers in their environments, more than half of the participants found it vital to learn to copresence with their peers in different ways—namely by reassembling their own bodies through the bodies of objects and gadgets, reconstituting themselves in physical or digital spaces that bridge geographical distance. In particular, gadgets become the only link between them and their peers. As Maddie put it: “I feel that [chatting and calling with my friends] is one way to keep me away from my anxiety with online class, like you’re all in this together even if you don’t see each other.” In the absence of physical bodies, these gadgets constitute the altered digital self: They become bodies in themselves, used to reach other bodies in space and time.

**Figure 9.**
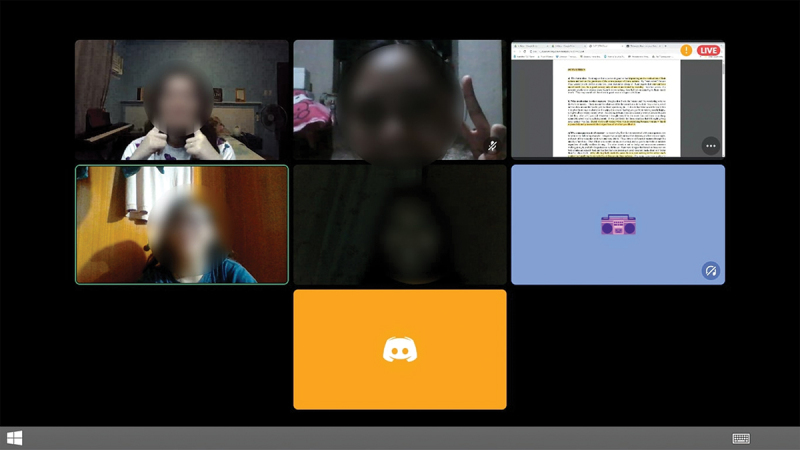
Copresencing with human others.

The ways by which participants recreated this sense of presence with their peers is particularly interwoven with the medium and digital platform used, making possible different kinds of copresencing. For example, a popular platform for many participants is Discord (see Figure 9). One participant, Riley, used Discord calls with her friends not only to interact with them through playing games but also to recreate the event of studying with her friends in the library. In Figure 9, the videos and voices of Riley’s friends through the screen and the speakers simulated the presence of other bodies; the shared ambient music played by the bot on Discord (radio icon) simulated the sense of being in a shared space; and the screen-sharing of Riley’s philosophy reading simulated the act of “studying” with her friends, allowing them to witness what she was working on to cultivate a sense of accountability. Thus, for the participants, the act of copresencing—being there for and with others—also means caring for the self.

**Figure 10.**
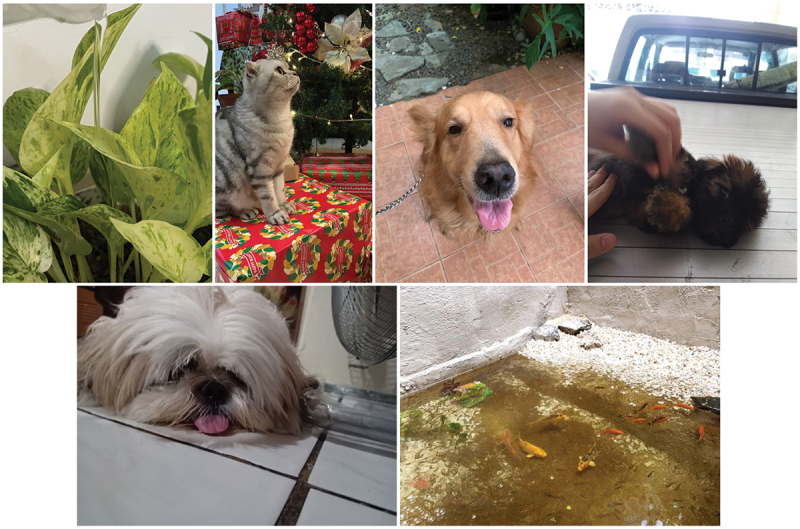
Copresencing with nonhuman others.

*Copresencing with nonhuman others*. About a third of the participants included photographs of their pets and plants during the interview (see Figure 10). For these participants, caring for the life around them is inextricably bound with self-care. As Grace put it, “Whenever I care for [my plants], it makes me care for me.” Contrary to human-centric accounts where “having pets” or “gardening” are considered forms of self-care to decrease stress and enhance wellbeing of the human actor, nonhuman actors here do not exist as passive entities upon which human beings benefit from; rather, the wellbeing of each folds into and prolongs into the other. There is no real separation between nonhuman and human life because they are intricately implicated in sustaining each other’s shared vitality.

While all participants mentioned that their pets or plants were already a part of their lives, over the course of the pandemic, there was a deepened sense of relationship with them just by their mere presence. Grace expressed it this way about the plant beside her desk in her room:

I like seeing my plants just there … I don’t know how one gets to know plants, but like there’s a certain energy they exude, like just seeing nature it makes you feel like, “Ahhhh (*sighs*), it’s okay.” Knowing there’s life next to me, it feels okay.

Copresencing assumes a proximal shared space: “Knowing there’s life next to me, it feels okay.” Here, part of caring for the self is also learning to simply be with nonhuman life and allowing themselves to be affected by the presence of that life. Within this cofunctioning, there exists an openness to the gentler intensities related to the vitality of pre-discursive, human-nonhuman copresencing. When before, some participants expressed feeling alive only when there were human beings around them, now, the mere presence of a plant or a beloved animal nearby helped them appreciate the unfolding of life, just as they are also witnessed by another life.

## Discussion

### The work-home assemblage as a fluid therapeutic assemblage

**Figure 11.**
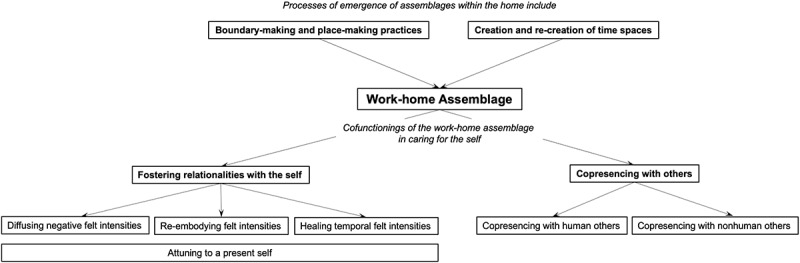
Summary of results.

By using assemblage thinking, the findings demonstrate the discursive-material complexity of how self-care practices are enabled within their homes during the pandemic (see Figure 11 for a summary of the results). Consistent with relational models of place (Conradson, 2005; Duff, 2012; Foley, 2011), certain places and spaces are not therapeutic in themselves but are made therapeutic through human and nonhuman interactions over time. Boundary-making and place-making practices allow the participants to differentiate pockets of wellness within their homes, where certain affective zones, such as one’s desk, are designated for schoolwork and stress; and zones like one’s bed and thinking space—cafe are designated for relaxation and reflection. New temporal rhythms were established by the creation and re-creation of time spaces guided by material objects, such as sticky notes or a calendar and to-do list. Together, these two processes co-mingled to form the makeshift work-home assemblage, which then functioned to enable practices of care primarily through fostering different relationalities with the self and through copresencing with human and nonhuman others.

These findings also show that the boundaries of the work-home assemblage are fluid and always-shifting (Law, 2004), in response to the different materials and objects that flow in and out of the spaces of the home. For example, different configurations of the work-home assemblage were used to foster different relationalities with the self: the yoga mat-phone-YouTube, assembled within an empty floor-space within a pocket of the home, helped participants diffuse negative felt intensities of stress and anxiety built throughout the day; the canvas-paint-brush meshwork (and the interviewer-interviewee-artwork meshwork) allowed participants to re-embody felt intensities; while the paint-brush-hand meshwork helped heal temporally discordant selves through time. To assemble these different practices from the work-home assemblage, participants had to attune to the myriad renderings of their current self. Thus, self-care practices are not derived from pre-existing, abstract categories (e.g., “physical”, “emotional”, “social”, etc.) (Lee & Miller, 2013) but rather are enabled “within the current of [the individual’s] life activities” (Ingold, 2000, p. 154) and in the contexts of the individual’s “practical engagement with their lived-in environments” (Ingold, 2000, p. 168). Rather than assuming self-care practices can be cognitively selected from a menu of different options, assemblage thinking allows us to view these practices as emerging from the meshwork of the individual’s relationships with the human, non-human, and material elements, forces, and affects in their life-spaces (Duff, 2014; Sultan, 2022).

This study also contributes to the existing strand of materialist psychological literature by providing a rich empirical account of using assemblage thinking to study psychological phenomena (Price-Robertson & Duff, 2016), such as the process of self-care. By using narratives and photographs as the multiple entryways into participants’ accounts (Sermijn et al., 2008), findings demonstrate how the self and its affective experiences are assembled through affective encounters with material objects, spaces, and time spaces, such as how Zoey’s abstract painting allowed her to re-embody her emotions and make sense of them during the interview. Additionally, the use of narratives enriches this understanding by allowing a glimpse into the ways that temporally discordant selves are encountered and re-embodied in the present and facilitate self-care through healing, as with the case of Lila’s painting of flowers on her skin. Thus, future studies could more productively explore rich accounts of participants’ experiences when the discursive, material, and temporal dimensions of their realities are considered through the assemblage.

### Self-care as an ethical embodiment of copresencing with the self and others

The pandemic is an ethical encounter of human and nonhuman “throwntogetherness” (Massey, 2005) wherein being confined into the space of the home calls into the question of how we live together in space. These encounters are facilitated by the body’s ability to establish connectivities with the spaces, objects, and nonhuman life—such as the yoga mat-YouTube video-yoga poses meshwork—which increased participants’ capacities to act. Expanding on the application of “care of self” as derived from Foucault’s work (e.g., Canoy & Ofreneo, 2017), what is salient in these accounts of caring for oneself is the sense of collaborative agency (McLeod, 2014) between human and nonhuman actors, and how material can be “agents of physical, social, and emotional movements” (Larsen et al., 2020, p. 6). Previously, participants expressed a taken-for-grantedness of the various activities that contributed to their wellbeing, such as physical activity and being around their peers; however, within the confines of the home, self-care and wellbeing had to be chosen and co-assembled with existing spatial and material resources.

Additionally, these findings emphasize that self-care is an embodied, temporal, and material process of being there for oneself and holding space for one’s own felt intensities. This is particularly important for young women, since studies have shown that women report experiencing both negative and positive emotions more intensely than men (Brebner, 2003). This being present for the self can also be understood in terms Law’s (2004) concepts of presences and absences. According to Law (2004), “What is being made present always depends on what is also being made absent” (p. 83)—there is an inherent relationality between the two. Prior to the pandemic, participants expressed how the various changing stimuli in the environments external to their homes distracted them from encountering themselves and their emotions. However, with the absence of physical spaces and the routines tied to these spaces during the pandemic, the presence of participants’ affective experiences became more immediately felt. The different discursive-material configurations of the work-home assemblage, then, were the various ways in which participants encountered their own different, embodied selves: These material and spatial practices, emerging organically from their environments, were what facilitated these healing encounters with their negative affective experiences, and what also helped them establish a sensibility of presencing with their different, dynamic selves.

During the pandemic, the absence of human others was also conspicuously experienced by participants. Pre-pandemic, participants’ worlds largely revolved around interacting with other human beings, particularly their peers—whether at school or in other places of recreation. Developmentally, this is also a period wherein most participants experience relationships with their peers to be the most crucial and formative to their identities (Rohrbeck & Garvin, 2003). Perhaps because of this, efforts of caring for the self also involve the active labour of reconstituting one’s own body and the bodies of others through online spaces. In other words, what seemed important to participants over verbal forms of human connection was the mere fact of knowing others are also “there” with them, experiencing what they were experiencing. Such an appreciation for presence only developed in participants through the manifest absence of others (Law, 2004). Following from this, the absence of the physical human bodies of their peers also made the presence of nonhuman life around them more deeply felt and appreciated—in the absence of others, the presence of other forms of companionship were reassembled within the work-home assemblage.

Finally, in assembling more facilitative intimate encounters with themselves, participants also experienced being able to be more fully present for others. Practices of copresencing with, and caring for, human and non-human others are inextricably intertwined with caring for the self. However, as a collectivist culture where emotional dependence from others is integral to one’s wellbeing and the wellbeing of the community (Uchida et al., 2008), there exists a prevailing moral discourse of self-care in the Philippines as “selfish” by giving in to one’s needs. While participants were aware of this, they instead resist this by positioning self-care as, in fact, integral to the service of others; and by casting self-care in relational terms—fostering a relationship with the self—thereby making this relationship of equal validity to one’s relationships with others.

## Limitations and future directions

This study demonstrates that Filipina university students, despite being vulnerable to the psychological impact of the pandemic, have the capacity to attend to their wellbeing and mental health through practices of self-care. A limitation of this study is its focus on relatively healthy, upper-middle-class, able-bodied female university students who have not yet experienced a family member having COVID-19 at the time of the study. Assembling practices of care are also necessarily limited to one’s existing material and spatial resources within the home, and in this upper-middle-class sample of university students seemed to have access to numerous art-related resources and private spaces within and outside of their homes, which may be different for students from lower socio-economic statuses. As such, future research may adopt an intersectional frame to investigate the different configurations of caring for the self and wellbeing in more diverse contexts, such as among different genders (for example, among male or queer/gender-nonconforming participants) or among people from different socioeconomic statuses.

## Conclusion

This paper has demonstrated the ways that combining narratives with the theoretical device of the assemblage can illuminate the ways that university students reinstitute self-care practices within the home during the pandemic. In particular, the relationalities of participants with a myriad of material and spatial arrangements through time facilitated participants’ relationalities and healing encounters with their own selves—their bodies, emotions, and past and future selves—and with the nonhuman others around them. Thus, the concept of “assemblage” shows that socio-material arrangements form organically, through living in particular spaces and through relationships with the objects around them; they are not something that arise in the participants’ minds and are subsequently imposed on reality. The invitation with thinking with assemblages, then, is to rethink the life-spaces where people’s actual self-care practices make are lived and enacted, grounded in their existing everyday realities of doing and being.

In conclusion, any intervention involving assemblage thinking is less about the imposition of a structured and instructional program of self-care, but rather more about cultivating a particular appreciation and sensibility towards the socio-material aspects of one’s everyday life. For example, with the transition to more hybrid forms of learning in a post-pandemic world, university counsellors and teachers may become more attentive to how these places of care form organically for students by asking what their sites of self-care are on- and off-campus and help enrich these spaces for the students. For example, perhaps one observes that students tend to congregate around the steps outside of the library to study together. University personnel can then include a nook for food and coffee for the students to address the need for sustenance while studying or provide a nook for donated bean bags or furniture to allow them to relax while having breaks. These examples demonstrate that assemblage thinking is about being attentive to existing everyday spaces and their uses, and about finding ways to re-assemble these spatial and material resources to enhance students’ mental health.
